# Case Report: Abdominal surgery with the support of Impella (SURGELLA), a new frontier to be explored

**DOI:** 10.3389/fcvm.2024.1301538

**Published:** 2024-04-04

**Authors:** Stefano Guarracini, Pierluigi Di Sebastiano, Fabio Francesco Di Mola, Raffaella Di Renzo, Lorenzo Mazzocchetti, Antonio M. Calafiore, Michele Di Mauro

**Affiliations:** ^1^ Department of Cardiology, “Pierangeli” Hospital, Pescara, Italy; ^2^ Unit of Surgical Oncology, Casa di Cura Pierangeli, Largo Luigi Pierangeli 1, Pescara, Italy; ^3^ Department of Innovative Technologies In Clinical Medicine & Dentistry, University “G. d'Annunzio” Chieti-Pescara, Chieti, Italy; ^4^ Department of Medical, Oral & Biotechnological Sciences, University “G. D'Annunzio” Chieti-Pescara, Chieti, Italy; ^5^ Department of Surgical Oncology, “Pierangeli“ Hospital, Pescara, Italy; ^6^ Department of Cardiac Surgery, Henry Durant Hospital, Athens, Greece; ^7^ Department of Cardio-Thoracic Surgery, Cardiovascular Research Institute Maastricht (CARIM), Maastricht, Netherlands

**Keywords:** heart failure, cancer, circulatory assist device, hemodynamics, impella

## Abstract

A 74-year-old man with advanced heart failure was admitted to the hospital with a diagnosis of colorectal cancer, and he underwent surgery. To maintain stable hemodynamics, the Impella CP device was used. The patient was weaned from the device shortly after surgery, and he had an uneventful postoperative course. This case may pave the way for non-procrastinating surgery in patients with poorly stable hemodynamics.

## Introduction

Colorectal cancer (CRC) is the third most common cancer in men and the second most common in women worldwide. In Italy, it is the second most common cancer (1). Recent studies have shown that the incidence rate of CRC in industrialized countries increases with age, as does the prevalence of concomitant diseases, especially cardiovascular (CV) disease. Therefore, with a longer life expectancy and an aging population, the number of patients with CRC and cardiovascular comorbidities is expected to increase (2). In a recent study, chronic heart failure (HF) was shown to increase the risk of 30-day mortality by up to 4-fold in patients with CRC undergoing colon resection (3).

It is now well-established that heart failure is a determinant of postoperative mortality and morbidity in a wide range of surgical specialties. In fact, it is included in all risk calculators for patients undergoing non-cardiac surgery (NCS) (4).

As a result, many patients are contraindicated for colon resection because of the very high perioperative risk, condemning them to an unfavorable evolution of CRC.

Here, we report a case that we hope will open the door to a multidisciplinary approach for patients with advanced HF who need to undergo colon surgery for the treatment of CRC.

## Case

A 74-year-old man with a history of anterior myocardial infarction who had already undergone coronary artery bypass surgery and several percutaneous interventions developed dilated cardiomyopathy with a clinical presentation of advanced HF and was admitted to our hospital with a diagnosis of CRC. The patient already had an ICD and CCM implanted. He also had chronic renal insufficiency (glomerular filtration rate of 35 ml/min), diabetes treated with insulin, and peripheral vasculopathy. On hospital admission, his blood pressure was 90 mmHg/50 mmHg, and therefore, he could not take angiotensin receptor–neprilysin inhibitor (ARNI), but he was on beta-blockers, sodium-glucose co-transporter (SGLT2) inhibitors, and mineralocorticoid receptor antagonists (MRA) at the maximum tolerated dose. In addition, a diuretic infusion had been started at home 1 week before hospitalization. Upon admission, the patient underwent a cardiological examination in accordance with the European guidelines (4). The results revealed that the patient had a high functional limitation (METS 2) with NYHA class IIIb. A physical examination showed mild bilateral peripheral edema with low blood pressure (90/60 mmHg) and a heart rate equal to 60 bpm. Echocardiography revealed that the left ventricular ejection fraction (LVEF) was approximately 15%, the tricuspid annular systolic plane excursion (TAPSE) was 12 mm, and the Brian natriuretic peptide (BNP) value was more than 30,000 pg/ml despite optimal drug therapy and diuretic infusion. The Revised Cardiac Risk Index for preoperative risk was 5 with an expected mortality rate of 15%; the percentages of the risk of major complications and cardiac complications according to American College of Surgery National Surgical Quality Improvement Program (ACS NSQUIP) were 40 and 16, respectively (4). The patient had been submitted to a hemodynamic evaluation four months before hospitalization with the following parameters: cardiac index = 1.91 L/min/m^2^, PAPm = 42 mmHg, wedge pressure = 28 mmHg, Pulmonary artery pulsatility index (PAPi) = 5.7, and right ventricular stroke work index (RVSWI) = 1050, Adx = 7 mmHg.

Given the hemodynamic instability and the development of tachyarrhythmias exhibited by the patient at the time of colonoscopy despite inotropic support, a multidisciplinary team opted for Impella CP with SmartAssist (Abiomed, Danvers, MA, USA), which is commonly used in our hospital to support patients during complex percutaneous coronary interventions.

The patient was taking aspirin, which was discontinued 5 days before the procedure and resumed 2 days after the surgical procedure.

First, the patient was lightly sedated to prevent his hemodynamics from getting affected. Then, the 14Fr Impella catheter was inserted at the level of the right common femoral artery.

A bicarbonate-based irrigation solution [sodium bicarbonate 8.4% 25 mEq in 1l dextrose 5% in water (D5W)] was used as an alternative to heparin in the Impella irrigation solution to reduce intraoperative and postoperative bleeding (5). After the Impella began to assist, general anesthesia was induced. A left laparotomy hemicolectomy with colostomy was successfully performed. The flow was set at 3.5 L/min.

Hemodynamic stabilization was maintained throughout the surgical procedure. Intraoperative blood pressure was 90/60 mmHg at the beginning of the operation and 100/60 mmHg at the end of the procedure. Intraoperative blood loss was 100 ml; the operation time of colectomy was 120 min, whereas Impella duration was 140 min; in-out volume balance was +500 ml.

The patient was provided three episodes of complete Impella support (first, during general anesthesia induction, second, when the patient was placed in the Trendelenburg position, and finally, just before the end of the procedure), demonstrating the need for Impella circulatory support. The patient was successfully weaned from the Impella, which was removed 10 min before extubation was done at the end of the surgical procedure; hemodynamics were stable during the postoperative course. After the removal of the Impella, the patient was provided inotropic support for 2 days with dopamine.

After this, he became fit for discharge, and he was discharged home after 1 week; his postoperative course was unremarkable. No bleeding or clotting events were recorded either during or after the surgical procedure. After the sixth month, the patient is alive with stable hemodynamics.

Written informed consent was obtained from the individual for the publication of any potentially identifiable images or data included in this article.

## Discussion

From this case report, we can derive some key points that obviously deserve to be confirmed in a large case series: (1) patients with end-stage cardiomyopathy and HFrEF can undergo life-saving surgical procedures, such as in cancer treatment; (2) the use of the Impella allows the surgeon to perform operations with hemodynamic stability without any fear of the side effects of inotropic therapy; and (3) multidisciplinary evaluation is essential, especially in this subset of patients.

Perioperative risk in non-cardiac surgery involves two main factors: the risk related to the type of procedure to be performed and the risk due to the patient's comorbidities (4). In our patient, both of these factors came into play: an increased perioperative risk was indicated, and indeed the surgical procedure was rejected by many surgeons previously.

In particular, our patient showed a very low left ventricular systolic function despite optimal medical therapy. This is noteworthy because the risk of adverse postoperative events associated with HF depends on three factors: whether LV systolic function is preserved or reduced, hemodynamic compensation, and the presence of symptoms (6). Patients undergoing NCS are at risk for acute decompensated HF with rapid onset or worsening of symptoms and/or signs of HF, triggered by fluid retention and/or comorbid conditions (7).

Of note, the risk of death increases progressively with decreasing systolic function. It is not recommended to perform elective NCS in patients with decompensated HF.

Therefore, inspired by the principles of Enhanced Recovery After Surgery (ERAS) (8), to increase the chances of recovery after surgery, we developed a multidisciplinary strategy aimed at improving perioperative treatment pathways to optimize their effectiveness and ensure faster patient recovery in the immediate postoperative period.

Among the various elements of ERAS, hemodynamic optimization represents a cornerstone that includes the use of fluids, vasopressors, and inotropics to ensure good pressure and blood flow throughout the procedure.

In our patient, the only way to keep hemodynamics stable during surgery was to use Impella CP. In the literature, it has been shown that the Impella has already been used to support hemodynamics in patients with abdominal aortic aneurysms undergoing vascular surgery (9) or as a bridging device until the implantation of a left ventricular assist device (10).

To our knowledge, this is the first case of a patient with CRC treated surgically using Impella. For this procedure, we have decided to coin the name SURGELLA (from Surgery and Impella).

However, further studies are certainly needed to demonstrate the benefits of Impella in high-risk patients undergoing non-deferrable surgery.

Other potential applications that remain to be explored could include leaving the Impella in place as a support to ensure adequate perfusion and hemodynamic stabilization even in the ICU to reduce postoperative complications and allow recovery (e.g., anastomotic leak, surgical site infection, renal insufficiency, and so on).

## Data Availability

The raw data supporting the conclusions of this article will be made available by the authors without undue restriction.
